# Deciphering Root Architectural Traits Involved to Cope With Water Deficit in Oat

**DOI:** 10.3389/fpls.2019.01558

**Published:** 2019-11-28

**Authors:** Francisco J. Canales, Kerstin A. Nagel, Carmen Müller, Nicolas Rispail, Elena Prats

**Affiliations:** ^1^ Institute for Sustainable Agriculture, Spanish Research Council (CSIC), Córdoba, Spain; ^2^ Institute of Bio- and Geosciences, IBG-2: Plant Sciences, Forschungszentrum Jülich GmbH, Jülich, Germany

**Keywords:** adult plants, drought, oat, rhizotron, root architecture, root morphology, root phenotyping

## Abstract

Drought tolerance is a complex phenomenon comprising many physiological, biochemical and morphological changes at both aerial and below ground levels. We aim to reveal changes on root morphology that promote drought tolerance in oat in both seedling and adult plants. To this aim, we employed two oat genotypes, previously characterized as susceptible and tolerant to drought. Root phenotyping was carried out on young plants grown either in pots or in rhizotrons under controlled environments, and on adult plants grown in big containers under field conditions. Overall, the tolerant genotype showed an increased root length, branching rate, root surface, and length of fine roots, while coarse to fine ratio decreased as compared with the susceptible genotype. We also observed a high and significant correlation between various morphological root traits within and between experiments, identifying several of them as appropriate markers to identify drought tolerant oat genotypes. Stimulation of fine root growth was one of the most prominent responses to cope with gradual soil water depletion, in both seedlings and adult plants. Although seedling experiments did not exactly match the response of adult plants, they were similarly informative for discriminating between tolerant and susceptible genotypes. This might contribute to easier and faster phenotyping of large amount of plants.

## Introduction

Water deficit is among the most important crop constraints, reducing quality, productivity and compromising economic output and food security worldwide (Farooq et al., 2009). Consequently, extensive research has been performed to elucidate the plant responses to water stress over the last decades (i.e. Flowers et al., 1977; Greenway and Munns, 1980; Franco et al., 2006; Osakabe et al., 2014). However, drought tolerance is a highly complex trait and despite the extensive past research efforts, the components contributing to tolerance remain poorly understood to date (Tuberosa et al., 2002). In addition, most research has focused on the impact of water stress on shoot development parameters, such as, leaf area and shoot dry weight, or agronomic traits such as yield whereas root traits have been largely neglected. The main reason for this is the technical difficulty to access and monitor whole root system through non-destructive and high-throughput phenotyping methods.

The root is the first organ exposed to the drying soil and the origin of the signals that orchestrate the machinery leading to drought tolerance (Schachtman and Goodger, 2008). A detailed study of root response to drought is therefore required to understand plant adaptation to water stress. Therefore, an expanding area of interest has been devoted, in recent years, to dissect root traits that may help crops to cope with water stress and maintain productivity under drought. The root system size, their properties and distribution ultimately determine the plant access to water, and hence, set the limits on the functioning of the aerial part of the plant (Comas et al., 2013). Different studies have shown drought-induced changes in several root architecture parameters (reviewed by Comas et al., 2013) but only a few compared well-characterized susceptible and tolerant genotypes under similar water stress deficit, including bean (Abenavoli et al., 2016), chickpea (Purushothaman et al., 2016), rice (Henry et al., 2016) and beech (Meier and Leuschner, 2008). This is crucial to discriminate changes that are simply related to cellular stress from those engaged to increase tolerance. The million-dollar questions are then which root traits may help the most in the selection of drought tolerant plants and under which circumstances these traits may be helpful.

Currently fast and accurate phenotyping is a “must” in breeding programs. Phenotyping seedlings under controlled conditions expedite the process of evaluation, which in case of root assessments is particularly welcome due to the inherent difficulties to access a system that operates in a below-ground environment. So far, most of the techniques developed to assess root architecture focused on seedlings. In some cases, root seedling phenotyping had some predictive value for later developmental stages, such as in Tuberosa et al. (2002) that showed a relation between seedling root traits and adult plant yield. However, in others the root phenotype of seedlings was not representative of that of adult plant (Watt et al., 2013). On the other hand, we have the challenge of extracting an entire root system from soil, while maintaining completeness and avoiding damage to the finest roots. Recently, some results have been generated on entire root systems through shovelomic approaches, but these seemed to be more appropriate for phenotyping crown roots than the whole root system (Lynch, 2011; Trachsel et al., 2011). For this reason, other root growth methods have been explored, such as the growth on moistened germination paper rolls or pouches (Watt et al., 2013; Gioia et al., 2017), the use of rhizotrons, in which the plants grow in a soil-filled chamber allowing the observation of root development (Nagel et al., 2012), and gel-based systems (Iyer-Pascuzzi et al., 2010) that allow digital recording of root architecture traits through scanners or digital cameras. For several researchers these methods are not an adequate solution yet, as they do not reflect the root system of adult plants growing under field or semi-field conditions (Smith and De Smet, 2012). This could be solved through X-ray computed tomography (Zhu et al., 2011) which has the capability to visualize root architecture *in situ* by collecting cross-sectional images and allows the 3D reconstitution of the root without damage. However, this method is not easily affordable for most research groups and/or for screening large amount of individuals. Thus, the big challenge is to set up a cultivation system that allow to dissect the most important traits contributing to drought tolerance, as early as possible to obtain reliable results reflecting what is happening in adult plants. The challenge is greater if we take into account that the solution may not be the same for each species.

Increased information regarding root system architecture can be found in model crops such as Arabidopsis (Dolan et al., 1993). However, greater efforts are necessary to dissect root architecture that contributes to cope with drought in cereals, since, in terms of root tissue organization, there are substantial differences between dicot models such as Arabidopsis and monocots. As far as we know, the majority of root architecture research in monocots has focused on maize and rice (Smith and De Smet, 2012).

Oat (*Avena sativa* L.) is an important crop ranking currently sixth in world cereal production (FAO, 2017). Although oats have vigorous root systems that exploit the soil well, their transpiration rates and water requirements are higher than that of other small grain cereals (Ehlers, 1989). In addition, oat are especially susceptible to grain abortion caused by drought, which shows as empty white spikelets (Sánchez-Martín et al., 2017). Therefore, there is a need to breed oat lines with higher yields under water-limited conditions. Recent studies have tackled the drought tolerance response of oat. These covered different aspects focusing on adaptation and yield potential (Sánchez-Martín et al., 2014; Sadras et al., 2017; Sánchez-Martín et al., 2018), on the role of different metabolites during drought tolerance responses (Sánchez-Martín et al., 2015; Sánchez-Martín et al., 2017; Gao et al., 2018; Canales et al., 2019), or on the identification of physiological and biochemical markers to select drought tolerant genotypes (Rabiei et al., 2012; Sánchez-Martín et al., 2012; Marcinska et al., 2017; Rispail et al., 2018). However, and following the same trend as for other crops, very few information are available regarding root responses engaged for drought tolerance in oat.

In this study, we aim to dissect the root system architecture components that contribute to oat’s ability to cope with drought by comparing two genotypes previously well-characterized under different water deficit conditions at field (Sánchez-Martín et al., 2014), physiological (Sánchez-Martín et al., 2012; Sánchez-Martín et al., 2015; Sánchez-Martín et al., 2018) and genetic levels (Montilla-Bascón et al., 2013). In addition, we investigated whether specific root traits assessed in seedlings grown either in pots or in rhizotrons, could reflect the adult plant’s reaction and hence facilitate screening of large amount of plants.

## Materials and Methods

### Plant Material

All experiments were carried out with the oat cultivars (cvs) Flega and Patones, which are susceptible and tolerant to drought stress, respectively (Sánchez-Martín et al., 2012; Sánchez-Martín et al., 2015; Sánchez-Martín et al., 2018). Patones exhibits a good adaptation to Mediterranean agroclimatic conditions. It was developed by ‘Instituto Madrileño de Investigación y Desarrollo Rural, Agrario y Alimentario’ (IMIDRA, Madrid, Spain), and ‘Plant Genetic Resources Center’ (INIA, Madrid, Spain) provided the seeds. Flega was developed by the Cereal Institute (Thermi‐Thessaloniki, Greece). Details of the genetic relationships between these cultivars have been previously reported (Montilla-Bascón et al., 2013) and showed that they are not closely related.

### Visual Assessment of Drought Symptoms

To confirm the genotype behavior under drought stress, drought symptoms were assessed in ten biological replicates per genotype/treatment according to Sánchez-Martín et al. (2018). Briefly, drought severity values were assessed daily according to a 0–5 scale where 0 = vigorous plant, with no leaves showing drought symptoms; 1 = one or two leaves show slight drought symptoms (less turgor) but most leaves remain erect; 2 = most leaves show slight levels of drought stress, however one or two leaves still show no drought symptoms; 3 = all leaves show drought symptoms but these are no severe; 4 = all leaves show severe drought symptoms including incipient wilting; 5 = the whole plant is wilted with all leaves starting to dry appearing rolled and/or shrunken (Sánchez-Martín et al., 2012). These data were used to calculate the area under the drought progress curve (AUDPC) similarly to the area under the disease progress curve widely used in disease screenings (Jeger and Viljanen-Rollinson, 2001) using the formula:

AUDPC = ∑ki=112[(Si+Si+1)(ti+1-ti)]

where S_i_ is the drought severity at assessment date i, t_i_ is the number of days after the first observation on assessment date i and k is the number of successive observations.

### Seedling Experiments

#### Pot Experiments

Experiments with seedlings growing in pots under controlled conditions were carried out as previously in our group (Sánchez-Martín et al., 2012; Sánchez-Martín et al., 2015; Sánchez-Martín et al., 2018) and also according to other researchers (Xiao et al., 2007; Hao et al., 2009; Gong et al., 2010). Ten biological replicates per cultivar and treatment were grown in 0.75 l pots (10 × 10 × 10.5 cm; one plant per pot) filled with peat:sand (2:1) sieved with a 3 mm grid, in a growth chamber at 20°C, 65% relative humidity and under 12 h dark/12 h light with 250 µmol m^−2^ s^−1^ photon flux density supplied by white fluorescent tubes (OSRAM, Garching, Germany). During growth, trays carrying the pots were watered regularly with tap water. After three weeks, water was withheld from drought-treated plants (Sánchez-Martín et al., 2012; Sánchez-Martín et al., 2015; Sánchez-Martín et al., 2018) for a period of 20 days producing a gradual soil water depletion. Control plants were watered as described earlier throughout the experiment. During the drought treatment, the relative soil water content (sRWC) was monitored daily, reaching a level of approximately 15–20% by day 18 which is consistent with previous drought-related studies on oat (Gong et al., 2010). This allowed us to confirm that during the whole drought time course Flega and Patones plants were subjected to similar soil relative water content and hence to similar stress dose as previously observed (Sánchez-Martín et al., 2012; Sánchez-Martín et al., 2015; Sánchez-Martín et al., 2018).

Sampling times were chosen to cover different levels of sRWC: still-sufficient water (6 days after water withholding (daww), 55–60% sRWC), mild water deficit (9 daww, 40–45% sRWC), moderate water deficit (12 daww, 30–35% sRWC), high water deficit (15 daww, 20–25% sRWC) and severe water deficit (18 daww; 15–20% sRWC). At each sampling times, roots of five oat plants per cultivar and treatment (well-watered and droughted) were harvested, washed out under tap water to remove soil residues and kept in 70% ethanol until used for morphological studies. At the latest time point plants were 38 days old.

#### Rhizotron Experiments

Rhizotron experiments were conducted at Jülich Plant Phenotyping Center (JPPC) (http://www.jppc.de) at Forschungszentrum Jülich GmbH, Germany. Oat seeds were imbibed in tap water for 3 h and pre-germinated at 20°C in wet tissue paper placed in Petri dishes in the dark for three days in climate chamber. Pre-germinated seeds with 2–4 mm radicle length were transferred into the rhizotrons (2 × 30 width × 60 depth cm) filled with 3 mm sieved potting soil adapted from Nagel et al. (2015). One seedling per rhizotron was placed 3 cm deep into the soil, embryo facing downwards positioned at the transparent surface of the rhizobox, which was thereafter covered with a black foil. The black cover was only removed for root growth measurements. After sowing, the rhizotrons were set in an angle of approximately 45°, with the clear face facing downwards.

The experiment was conducted in a complete randomized design including two oat cultivars (Flega and Patones), two water treatments and six biological replicates per genotype and treatment. The water treatments were: (1) high water deficit (HWD, 20% field water capacity, FWC) and well-watered (WW, 90% FWC). Soil for HWD treatment was dried in an oven to reach 20% of sRWC. Control plants were watered regularly with tap water. After transferring the seedling, 10 ml of tap water was added to the soil in drought treatment nearby to the pre-germinated seeds to help plant establishment at the beginning. Water content of the soil was checked three times per week by weighing the rhizotrons in order to confirm that the sRWC of each treatment was maintained throughout the experiment. The position of the rhizotrons in the growth chamber was randomly changed at each measurement time point to reduce the effect of local differences in environmental conditions (such as temperature and air humidity). Plants were grown at 20°C, 65% relative humidity and under 12 h dark/12 h light in the climate chambers of the Institute for Plant Sciences (IBG-2; Forschungszentrum Jülich GmbH, Jülich, Germany). The drought treatment was performed according to the established protocols at IBG-2 (for more details see Nagel et al., 2012; Avramova et al., 2016). The experimental settings were different from the pot experiments due to rhizotron particular requirements but drought symptoms in the susceptible and resistant genotype followed a similar pattern.

Three times per week, the length and width of all leaves were measured with a ruler. The measurement started 6 days after sowing, when the first leaf was unrolled. The total leaf area (A) was then calculated according to A = leaf width × leaf length × 0.858 (Kalra and Dhiman, 1977). The measurement of roots started 6 days after sowing, when the first roots were visible at the transparent surface of the rhizotrons. Three times per week a digital image of the visible roots at the transparent window was taken, and length of the root, root system width, convex hull area, and seminal root length were calculated with RhizoPaint (Nagel et al., 2012). Experiment ended 30 days after sowing once control plant roots of both genotypes reached the bottom of the rhizotrons.

The visible root length at the surface of the rhizotron represented a portion of the total root system length. To establish the effect of different treatments on whole root length, it was necessary to define the relationship between visible and non-visible roots. To do this, plants were harvested at the end of the experiment, washed out under tap water, and total root length was determined with WinRHIZO (Regent Instruments Inc., Québec City, QC, Canada). The visible root length represented approximately 18% of the total root length, which is consistent with previously published data (Hurd, 1964; Nagel et al., 2012). The root length visible at the rhizotron surface and the total root length (visible and non-visible roots) showed a strong correlation with r^2^ = 0.95 (*P <*0.001). The ratio between visible and non-visible roots was unaffected by treatment and no differences between control plants of the two cultivars were found.

### Adult Plants Experiments

Nine replicated oat plants per cultivar and treatment were sown in big containers (27 × 27 × 45 cm), one plant per container, in order to be able to recover the entire root system. Containers were placed outdoor under climatic field conditions at Institute for Sustainable Agriculture in Córdoba, Spain (N37°51′38.1″ W4°47′40.8″). Containers were filled with approximately 20 kg of a mixture of peat:sand (2:1) sieved at 3 mm. Plants were sown on 15th December. During growth, containers were watered regularly with tap water. Plants were fertilized by foliar spray of Microsolem^®^ according to manufacture instructions at tillering and panicle emergence. At the beginning of tillering (stages 21–22 according to Zadoks scale, (Zadoks et al., 1974), all plants were watered to saturation, left to drain for 2 h and then the top of the container was covered with a transparent film with a hole for the shoots to prevent evaporation. This allowed a slow and gradual depletion of the soil water content mimicking the terminal drought characteristic of the Mediterranean area. When rain was foreseen, plants were protected by a transparent plastic awning that was removed immediately after rain. As stated above this was not often and plants were protected from short showers only five times. During the drought treatment, the RWC of the soil in the containers was monitored daily, reaching a level of approximately 15% after 31 days. Control plants were watered regularly throughout the whole experiment. As an additional control of growth and phenology and to confirm that, the containers did not limit the growth of oat plants. Flega and Patones seeds were also sown in the nearby field with a randomized complete block design with three biological replicates. Each replicate consisted of independent plots sown in three 1-m-long row at a density of 90 seeds m^−2^. Within each plot, rows were separated from each other by 50 cm. Control plants growing in the containers showed similar phenology and they did not show any restriction in their growth compared with plants growing in the field trial. Plants growing in the containers even showed a slightly higher yield probably due to the differences in the soil composition and structure and lack of competition.

At the beginning of ripening, [stage 90 of Zadoks scale (Zadoks et al., 1974)] the number of tillers, stem number and number of active leaves/stem were recorded. At the end of the experiment, when plants were approximately 4 month-old, seed weight and total aerial dry mass were recorded. In addition, the roots of each plant were harvested, washed out and stored in plastic bags filled with 70% ethanol at 4°C for morphological studies (Meyer et al., 2009).

### Leaf Chlorophyll Content

Leaf chlorophyll was indirectly estimated on the adaxial side of the leaf of oat adult plants using a SPAD-502 chlorophyll meter (Minolta Co., LTD., Japan). Plant SPAD index was calculated as the mean of all leaves. To obtain the index for one leave, three different measurements in the tip, mid and base of the leaf, were recorded and averaged. Measurements were taken 6 h after the onset of the light period at the beginning of ripening [stage 90 of Zadoks scale, (Zadoks et al., 1974)].

### Stomatal Conductance

Leaf water conductance was measured with an AP4 cycling porometer (Delta-T Devices Ltd, Cambridge, UK) according to Prats et al. (2006). Measurements were carried out in the flag leaf of each stem 6 h after the onset of the light period at the beginning of ripening [stage 90 of Zadoks scale, (Zadoks et al., 1974)].

### Morphological Root Trait Assessment

Root fixed in 70% ethanol were stained with an abundant volume of 0.01% neutral red (Sigma Chemical Co.) during 24 h to increase contrast in the image staining solution (Schumacher et al., 1983). The stained roots were placed in a transparent tray with a thin layer of water and scanned at a resolution of 600 pixels per mm. Root images were analyzed using WinRHIZO (Regent Instruments Inc., Québec City, QC, Canada) as described by Himmelbauer et al. (2004). Total root length, root surface, root diameters, number of tips and branches, length of fine (<0.5 mm) roots, and the coarse (>0.5 mm) to fine root ratio were recorded.

To scan the roots of adult plants, roots were trimmed in approximately 5 cm long fragments and homogenized. Note that for scanning the whole root system of one adult plant up to 80 trays were necessary to be scanned. In addition we tested whether root parameters of the entire root system could be extrapolated on the basis of the measurements of six subsamples for which six random selected subsamples of the whole root system were scanned and weighted. The method was validated for each cultivar and treatment separately. Based on these data different regression curves (for the different root parameters) were derived. To validate the system five replications (selecting different sets of subsamples) for each genotype and treatment were performed. In all cases r^2^ ranged from 0.97 to 0.99 and the coefficient of variation between the estimated size of whole root system predicted by the different subsample sets was always lower than 5%. Consequently, the regression curves obtained from each cultivar and treatment were used to determine the root parameters of the corresponding replications by scanning each six subsamples. Scanned roots were dried in a forced-air dryer for 24 h at 75°C in order to determine root dry weight.

### Statistical Analysis

For statistical analysis, data recorded as percentages were transformed to arcsine square roots (transformed value = 180/п × arcsine [√(%/100)]) to normalize data and stabilize variances throughout the data range. However, for ease of understanding means of raw percentage data are presented in figures. Data obtained from non-destructive sampling (i.e. drought symptoms or rhizotron measurements) were subjected to Repeated Measurement analysis using SPSS software (IBM, SPSS). The between-subject main effect was genotype and the within-subject or repeated measures effect was time of measurement. Homoscedasticity of residuals was confirmed by Box’s test and Greenhouse–Geisser epsilon accounted as correction factor. Data obtained from destructive sampling were subjected to Two-way ANOVA, with genotype and time as factors, using SPSS software (IBM, SPSS) and residual plots were inspected to confirm normality of data. Significance of differences between means was determined by contrast analysis (Scheffe’s). For multivariate analysis, data were analyzed using principal components analysis (PCA; (Causton and Causton 1987)) and cluster analysis using PAST free software 3.22 (https://folk.uio.no/ohammer/past/). Pearson correlations were calculated to detect statistical correlations between traits.

## Results

### Root Morphological and Architectural Changes During Water Deficit in Seedlings

#### Pot Experiments

Several independent water deficit experiments were carried out to determine the morphological and architectural changes that are responsible for the tolerant phenotype of cultivar Patones in contrast to the susceptible Flega. As previously observed (Sánchez-Martín et al., 2012; Sánchez-Martín et al., 2015; Sánchez-Martín et al., 2018), the sRWC reduced exponentially during the drought time course in all experiments. The sRWC decrease followed the same progression curve for both cultivars indicating that they were subjected to similar water stress throughout the experiment. The last harvesting was carried out at 15–20% sRWC with Flega plants suffering severe drought symptoms, such as loss of turgor and chlorosis, albeit still far from the wilting point. Flega and Patones plants showed similar phenology although drought related symptoms initiated earlier and were more severe on Flega than on Patones (**Supplementary Figure 1**). Both genotypes reduced the growth of leaves compared with non-stressed well-watered controls (*P < *0.001) but droughted Patones plants maintained higher leaf area and leaf dry matter than the susceptible Flega (**Supplementary Figure 1**)

**Figure 1:**
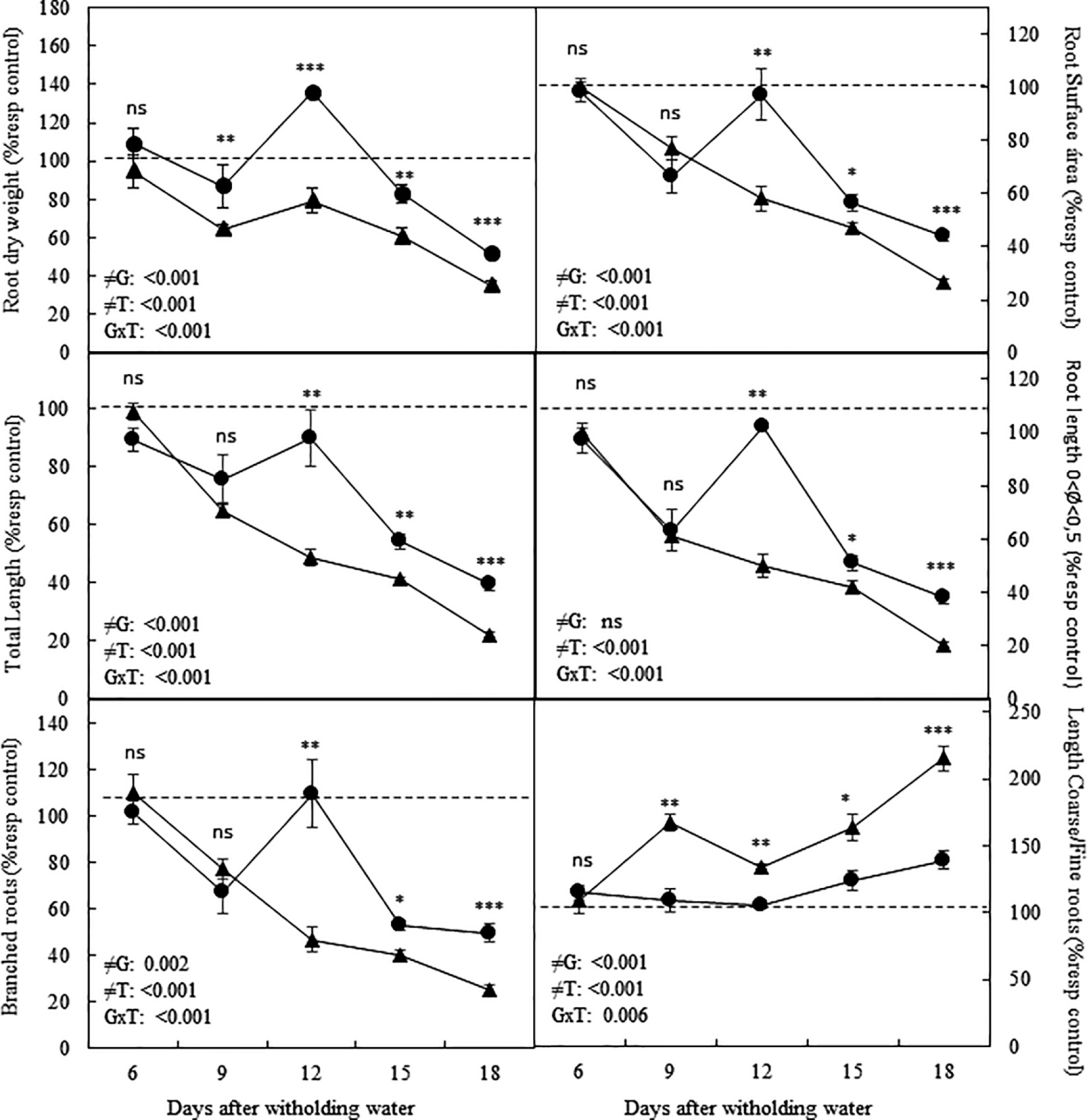
Root morphological and architectural-related traits of oat seedlings grown in pots during a water deficit time course. Data of Flega (triangles) and Patones (circles) plants are expressed as percentage with respect to control plants growing in well-watered conditions and are mean of ten biological replicates ± standard error. ≠G, ≠T and GxT indicates statistical significance for the time course between genotypes (G), sampling times (T) and their interaction, respectively. The dashed line indicate the performance of well-watered plants. *, **, *** indicate significant differences at P < 0.05, 0.01 and 0.001, respectively, ns indicates non-significant differences.

Drought treatment also significantly affected root growth as compared with non-stressed plants (*P < *0.001 for all traits assessed). A detailed analysis of the root morphological changes occurring over the drought time course showed that most traits followed a similar pattern (**Figure 1**). Root dry weight, total root length, root surface area and the number of branched fine roots tended to be lower under water deficit in both genotypes compared to well-watered plants from 9 daww onward. However, the tolerant genotype, Patones, presented significantly higher values for any of the above-mentioned root morphological traits, than the susceptible Flega (**Figure 1**). Interestingly, the length of fine roots (diameters between 0 and 0.5 mm) followed a similar decreasing trend as the total root length indicating the importance of these fine roots in the total root length. Drought treatment significantly reduced the average root diameter of both genotypes (*P < *0.001). However, the ratio between coarse roots (Ø > 0.5 mm) and fine roots (Ø < 0.5 mm) was significantly lower in droughted Patones plants than in Flega plants, except for the earliest sampling time. This suggested that the root growth observed in Patones under drought was mainly due to the production of fine roots (**Figure 1**). This would be in accordance with the higher number of fine roots observed in Patones as compared with Flega. Thus, despite the reduction in root growth rate detected in both cultivars in response to drought, the tolerant genotype maintained a higher root growth rate than the susceptible cultivar, producing mainly fine roots. The correlation between the different morphological traits assessed was very high with r^2^ of 0.98–0.99 (*P < *0.001, **Table 1**).

**Table 1:** Pearson correlations between root architectural parameters: number of branched root, total length and root surface of Flega and Patones seedlings growing in pots and rhizotrons during a water deficit time-course.

	Length fine roots	Branched roots	Total length
Pots			
Branched roots	0.9881***		
Total Length	0.9998***	0.9868***	
Surface	0.9965***	0.9810***	0.9979***
			
Branched roots	0.9360***		
Total Length	0.9978***	0.9379***	
Surface	0.9802***	0.9342***	0.9904***

***indicate significant differences at P < 0.001.

#### Rhizotron Experiments

**Figure 2:**
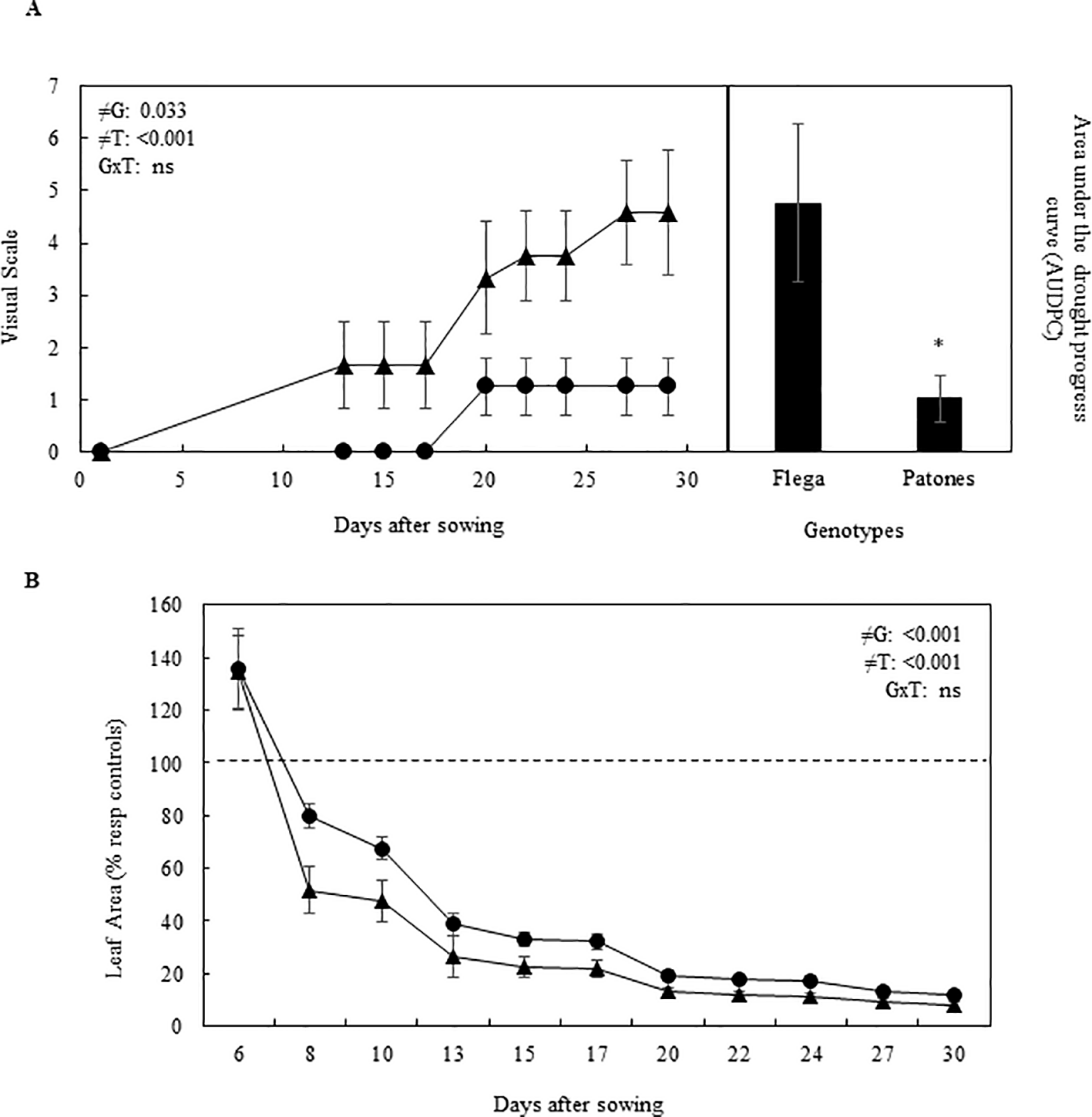
Effect of water deficit on oat seedlings grown in rhizotrons during a time course **(A)** Drought symptoms and **(B)** Leaf area of Flega (triangles) and Patones (circles) oat plants. Leaf data are expressed as percentage respect to control plants growing in well-watered conditions. All data are mean of ten biological replicates ± standard error. ≠G, ≠T and GxT indicate statistical significance for the time course between genotypes (G), sampling times (T) and their interaction, respectively. The dashed line indicate the performance of well-watered plants. *, indicate significant differences at P < 0.05.

Although drought symptoms developed slower in rhizotron than in pots, they initiated earlier and were more severe in Flega than in Patones (**Figure 2**), as expected. Leaf area of both Flega and Patones were reduced under drought compared with well-watered controls from day 8 after sowing. However, the drought-induced reduction in leaf area was lower in Patones than in the susceptible Flega (P = 0.04, **Figure 2**).

**Figure 3:**
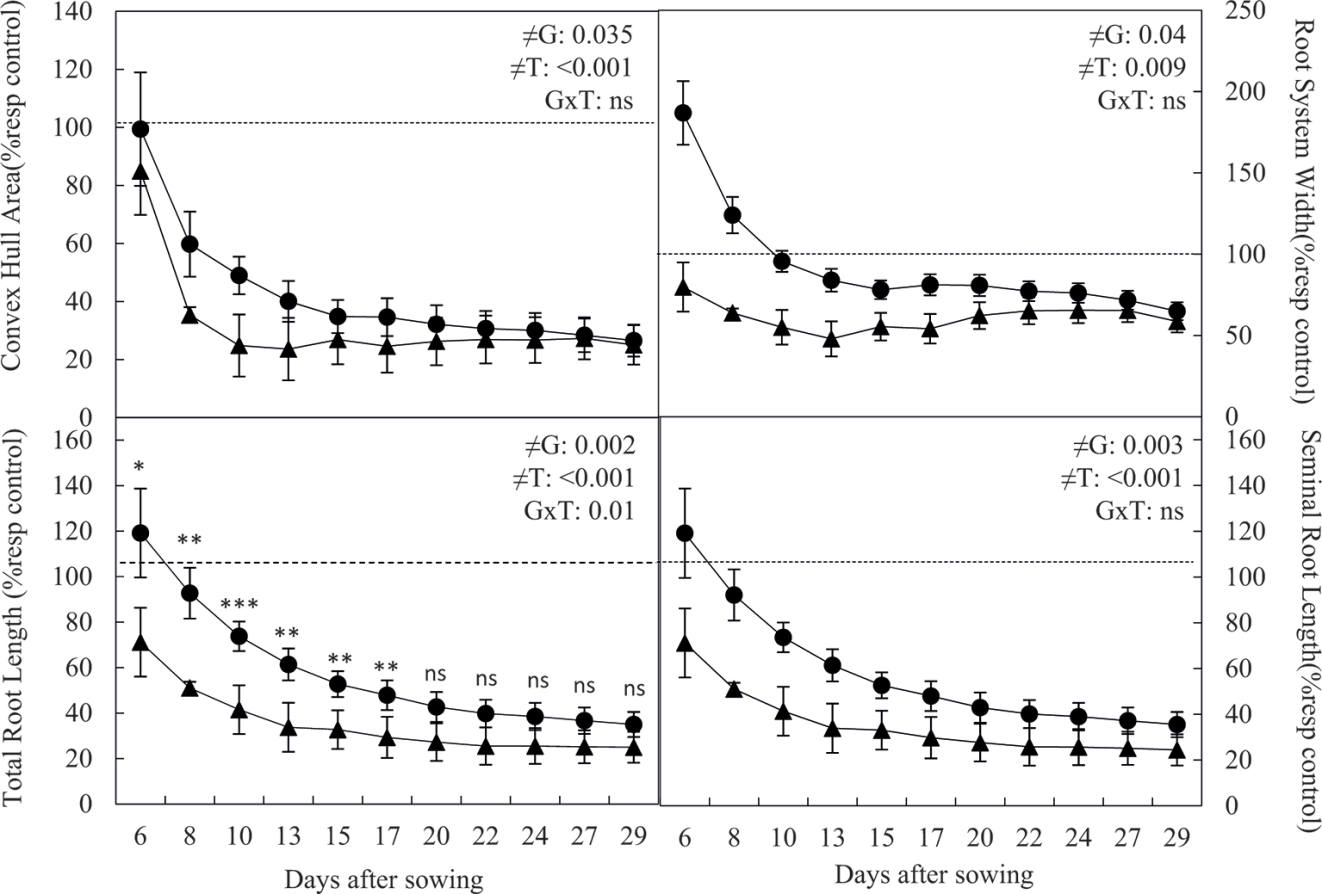
Root morphological and architectural-related traits of oat seedlings grown in rhizotrons during a water deficit time course. Data of Flega (triangles) and Patones (circles) are expressed as percentage with respect to control plants growing in well-watered rhizotrons and are mean of ten biological replicates ± standard error. ≠G, ≠T and G × T indicate statistical significance for the time course between genotypes (G), sampling times (T) and their interaction, respectively. The dashed line indicate the performance of well-watered plants. *, **, *** indicate significant differences at P < 0.05, 0.01 and 0.001, respectively, ns indicates non-significant differences.

**Figure 4:**
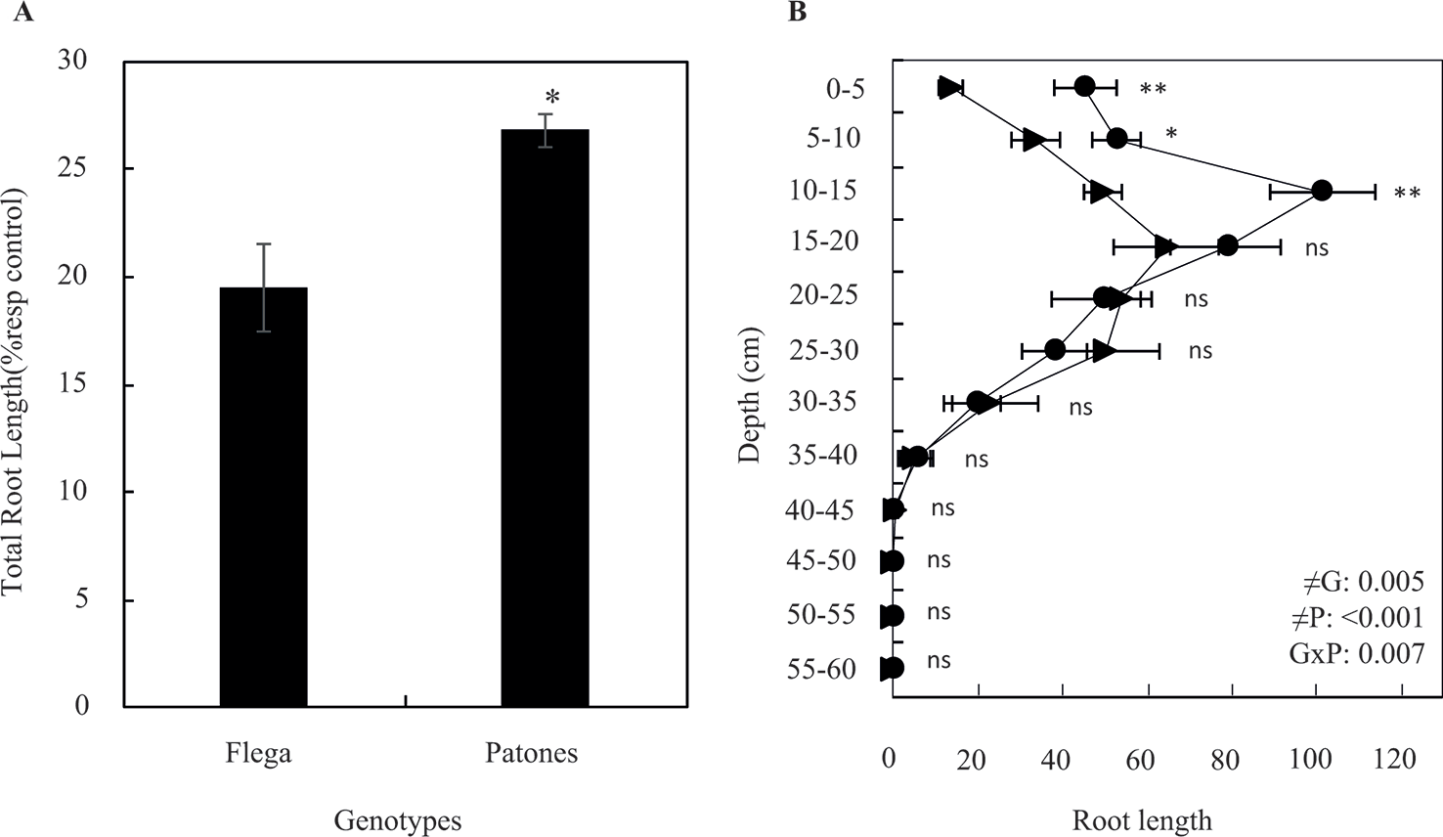
Total root length **(A)** and its distribution in depth **(B)** of oat seedlings grown in rhizotrons during a water deficit time course. Data of Flega (triangles) and Patones (circles) are expressed as percentage respect to control plants growing in well-watered rhizotrons and are mean of ten biological replicates ± standard error. ≠G, ≠T and G × T indicate statistical significance for the time course between genotypes (G), sampling times (T) and their interaction, respectively. *, **, indicate significant differences at P < 0.05, and 0.01, respectively, ns indicates non-significant differences.

Analysis of the rhizotron images, taken periodically over the 30 days of the experiments, allowed recording additional morphological and architecture root parameters including convex hull area or root width. Water stress reduced in both cultivars the convex hull area, which is the area covered by a root system measured by encompassing a root system with the shortest line (**Figure 3**). However, the convex hull area of Patones plants under drought was significantly higher than that of Flega (*P* = 0.03). The root system width, which is the maximal horizontal distribution of the root system, followed a similar trend. Accordingly, water stress reduced the root width of both cultivars, although it remained higher in Patones than in Flega (*P < *0.001). Similar to the pot experiment, water stress reduced the root growth rate of both Flega and Patones grown in rhizotrons as shown by the reduction in the seminal root length and whole root length of water stressed plants compared to well-watered plants. Nonetheless, the length of both seminal root and whole root system was higher in droughted Patones than in droughted Flega (*P* = 0.003 and 0.002, respectively). Analysis of root images according to the different soil profiles showed that most roots were produced in the first 40 cm with the highest density at 15–20 cm (**Figure 4**). No differences in rooting depth were observed between Flega and Patones. Scanning of roots at the end of the rhizotron experiments confirmed that Patones plants had longer roots under water stress than Flega (*P < *0.05). In addition a correlation of r^2^ = 0.9512 (*P < *0.001) was observed between the data obtained from rhizotron images and scanned roots indicating that under the conditions used the visible roots at the transparent rhizotron surface are a good representation of the total plant root system.

**Figure 5:**
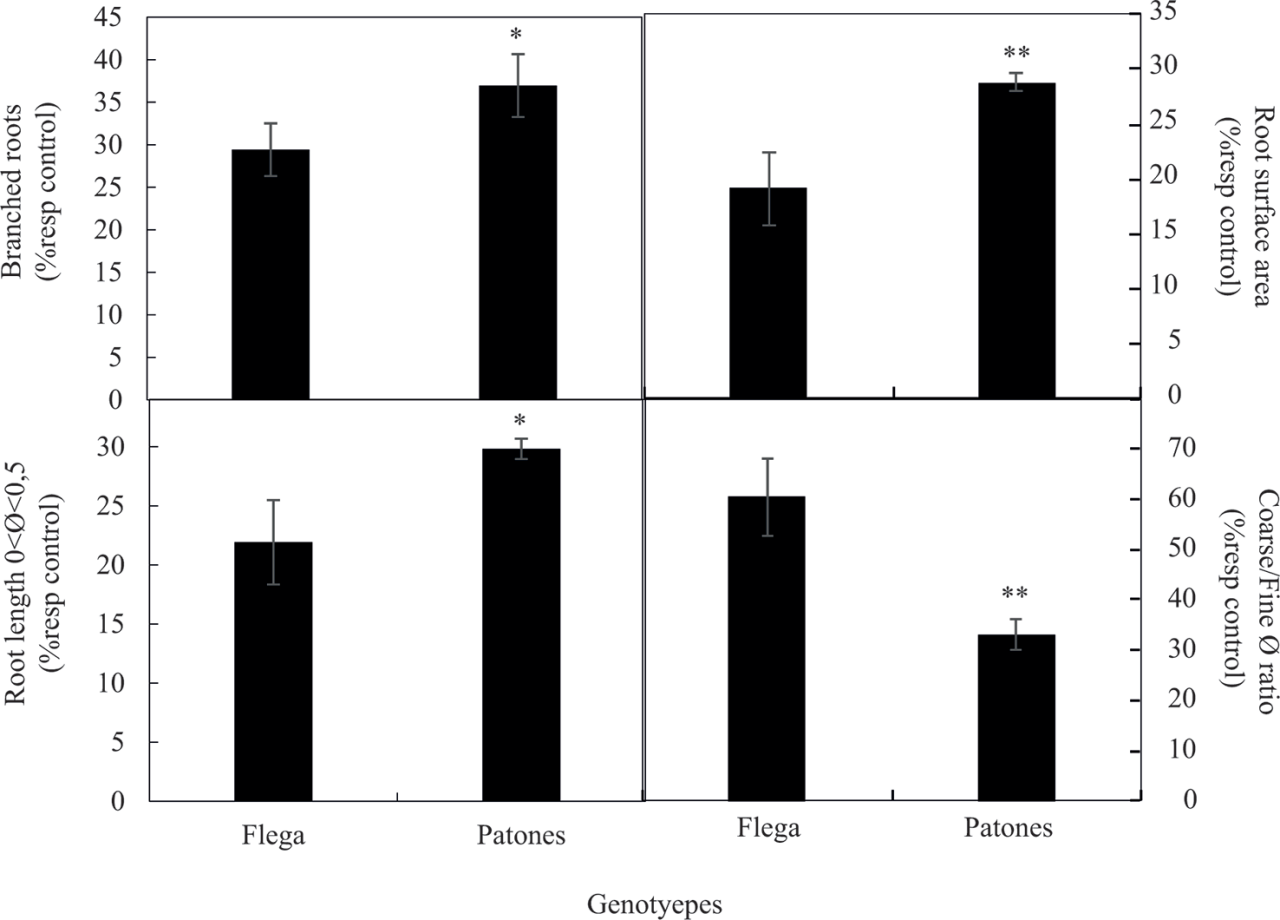
Root morphological and architecture related traits of oat seedlings after finalizing its growth in rhizotrons subjected to water stress. Data of Flega and Patones are expressed as percentage respect to control plants growing in well-watered rhizotrons and are mean of ten biological replicates ± standard error. *, ** indicate significant differences at *P < *0.05 and 0.01, respectively.

Scanning roots at the end of the rhizotron experiments allowed recording similar morphological data as the pot experiments. We observed a similar trend in rhizotron and pot experiments, with the tolerant Patones plants showing significant increased root surface area, branching, and length of fine roots together with a reduced coarse/fine root ratio when compared with Flega plants under drought (**Figure 5**). These morphological traits were highly and significantly correlated with coefficient of correlation (r^2^) ranging between 0.93 and 0.99 (*P < *0.001).

### Root Morphological and Architectural Changes During Water Deficit in Adult Plant

**Figure 6:**
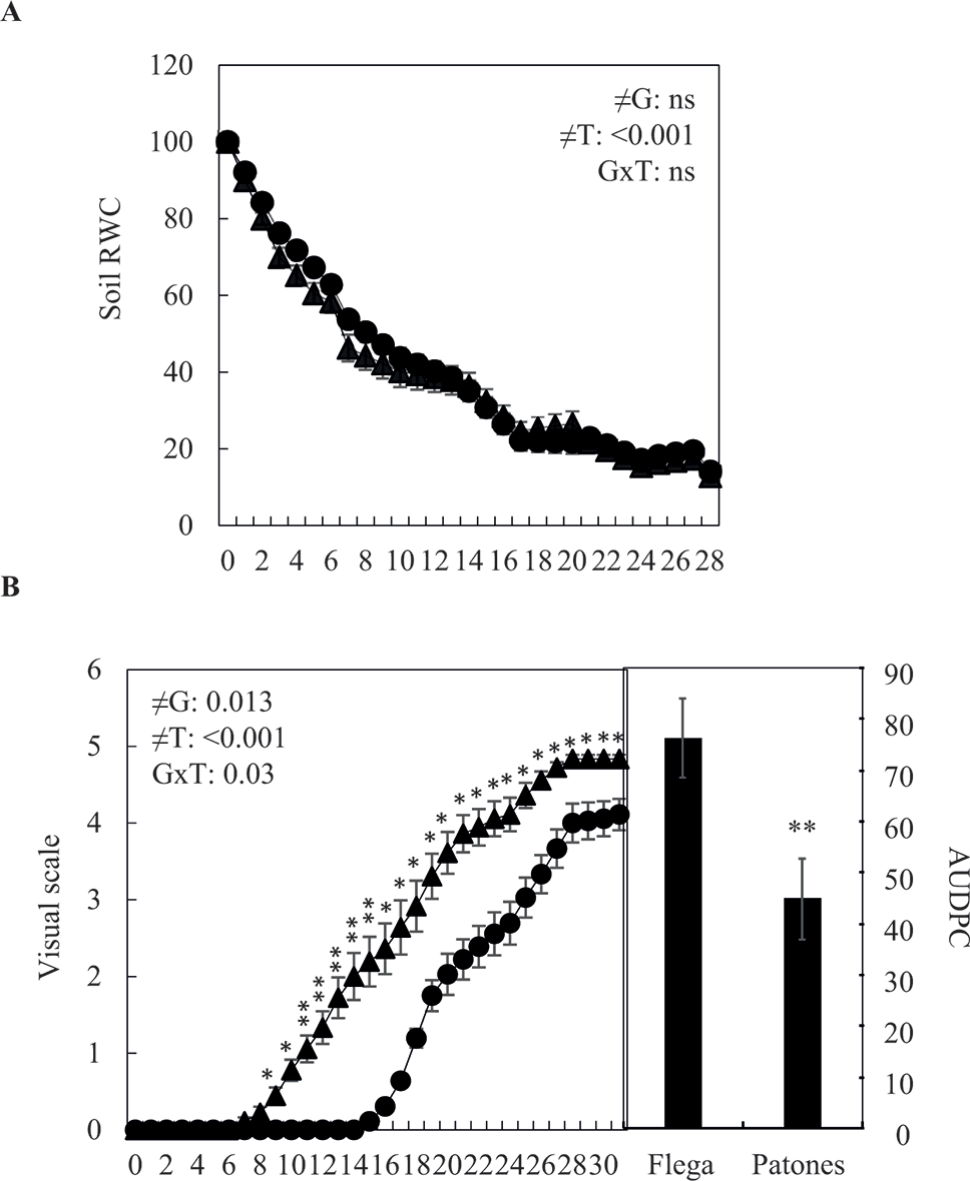
Effect of water deficit on oat adult plants grown under climatic field conditions **(A)** Soil relative water content and **(B)** Drought symptoms of Flega and Patones adult plants during a drought time course. Data are expressed as percentage respect to control plants growing in well-watered conditions and are mean of eight biological replicates ± standard error. ≠G, ≠T and G × T indicate statistical significance for the time course between genotypes (G), sampling times (T) and their interaction, respectively. AUDPC indicate the cumulative values of the area under the drought symptom progress curve. *, **, indicate significant differences at *P < *0.05, and 0.01, respectively, ns indicates non-significant differences.

Measurements of the soil RWC confirmed that both, Flega and Patones were subjected to similar water stress throughout the experiment (**Figure 6A**). As previously observed in the seedling experiments (Sánchez-Martín et al., 2015), well-watered adult plants of Flega and Patones showed similar phenology and development (**Supplementary Table 1**). This included a similar flowering time as previously observed in several field trials in the Mediterranean area (Sánchez-Martín et al., 2014). Adult plants of Flega showed earlier and more severe drought symptoms than Patones (*P* = 0.013). This was reflected by the significantly lower AUDPC of Patones plants (**Figure 6B**). Water stress also led to an earlier senescence of Flega leaves that showed approximately 90% of dead leaves at 31 daww compared with the 70% of Patones (**Supplementary Figure 2**)

A detailed analysis of the gradual soil water depletion effect on adult oat plants confirmed Flega as more susceptible than Patones. Although the number of tillers was not significantly different between cultivars, drought-stressed Patones showed significantly higher number of stems and active leaves per stem with respect to their well-watered controls than Flega (**Supplementary Figure 3**). The relative seed weight per plant and total dry weight was also significantly higher in Patones. Furthermore, Patones SPAD index that estimates the leaf chlorophyll content, was also higher than in Flega suggesting these plants maintained a higher photosynthetic status (**Supplementary Figure 3**). Interestingly, no differences were found in SPAD index or stomatal conductance between droughted Patones and Flega plants when considering only flag leaves (**Supplementary Figure 4**)

**Figure 7:**
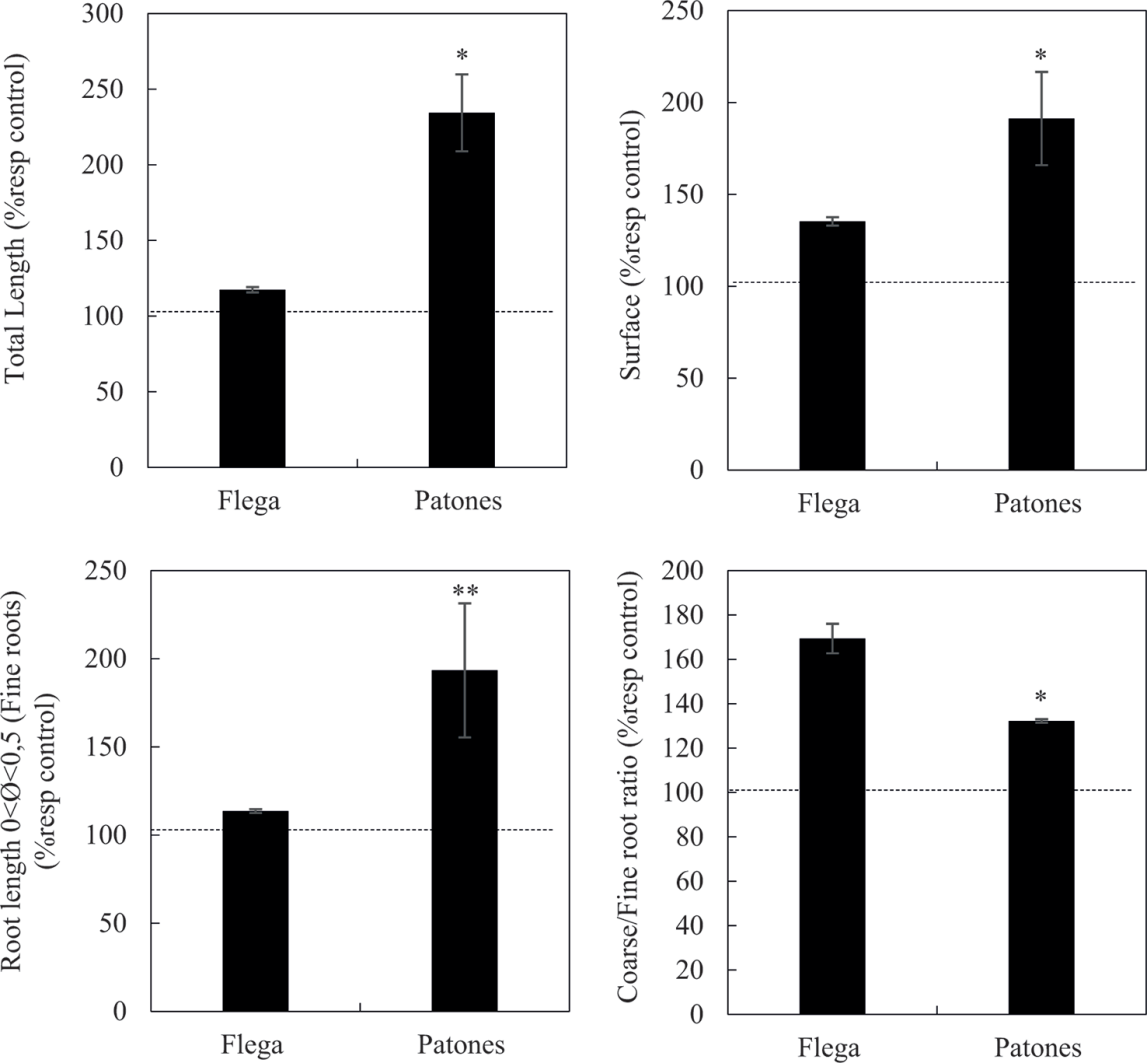
Root morphological and architectural-related traits of Flega and Patones adult plants under field conditions during a drought time course. Data are expressed as percentage respect to control plants and are mean of four biological replicates ± standard error. *, ** indicate significant differences at *P < *0.05, and 0.01, respectively. The dashed line indicate the performance of well-watered plants.

Assessment of the root morphological traits showed that Patones plants developed a longer root system than Flega (**Figure 7**). By contrast, to the observation in seedlings, the root systems of adult plants subjected to drought were higher than that of control plants. The growth difference between droughted and well-watered control plants was small for Flega but it was more than 2-fold for Patones. As previously observed in seedlings, this change was mainly due to an increase in the fine root portion of the root, with diameters between 0 and 0.5 mm (**Figure 7**). Thus, the drought-induced changes in root morphology followed the trend previously observed in seedlings. Accordingly, the coarse/fine root ratio was smaller in the tolerant Patones indicating that this cultivar developed a higher proportion of fine roots (**Figure 7**). Root surface area was also higher in droughted plants compared to their well-watered controls with Patones showing a higher root surface than Flega. The different root traits assessed in adult plants were significantly correlated between them with correlation coefficient ranging between 0.90 and 0.99 and *P < *0.001 (**Table 2**).

**Table 2:** Pearson correlations between root architecture parameters: number of branched root, total length and root surface of Flega and Patones adult plants growing under field conditions during a drought time course.

	Length fine roots	Total length
Length	0.9967***	0.9174***
Surface	0.9020***	

***indicate significant differences at P < 0.001.

### Correlation of Root Traits Between Pots, Rhizotrons and Adult Plant Experiments

To determine whether the root trait changes observed in young plants during the drought stress experiments were representative of those observed in adult plants grown in container we performed Pearson correlations. Data showed that all assessed parameters were correlated between all experiments including total root length, length of fine roots and root surface. The highest correlation coefficients (r^2^ from 0.84 to 0.87; *P < *0.001, **Table 3**) were observed between pots and rhizotron experiments. Root traits of adult plants showed a highly significant negative correlation with seedlings because root growth was stimulated in drought-stressed adult plants compared with their controls. However, the differences detected between susceptible and tolerant plants under water stress at adult and seedling stages were similar. The highest significant correlation was found between root traits of adult plants with those recorded from rhizotron experiments with r^2^ = −0.62, −0.59 and −0.63 for total root length, length of fine roots and root surface, respectively. Correlation between the root traits of adult plants and those from seedlings grown in pots were also significant but presented slightly lower coefficients (**Table 3**).

**Table 3:** Pearson correlations between root architecture parameters: total length, length of fine roots and root surface of Flega and Patones seedlings grown in pots and rhizotrons and adult plants grown under climatic field conditions during a drought time course.

	Total length		Length fine roots		Root surface	
Rhizotrons	−0.62**		−0.59**		−0.63***	
Pots	−0.46*	0.85***	−0.44*	0.84***	−0.56**	0.87***
	Adult	Rhizotrons	Adult	Rhizotrons	Adult	Rhizotrons

*, **, *** indicate significant differences at P < 0.05, 0.01 and 0.001, respectively.

**Figure 8:**
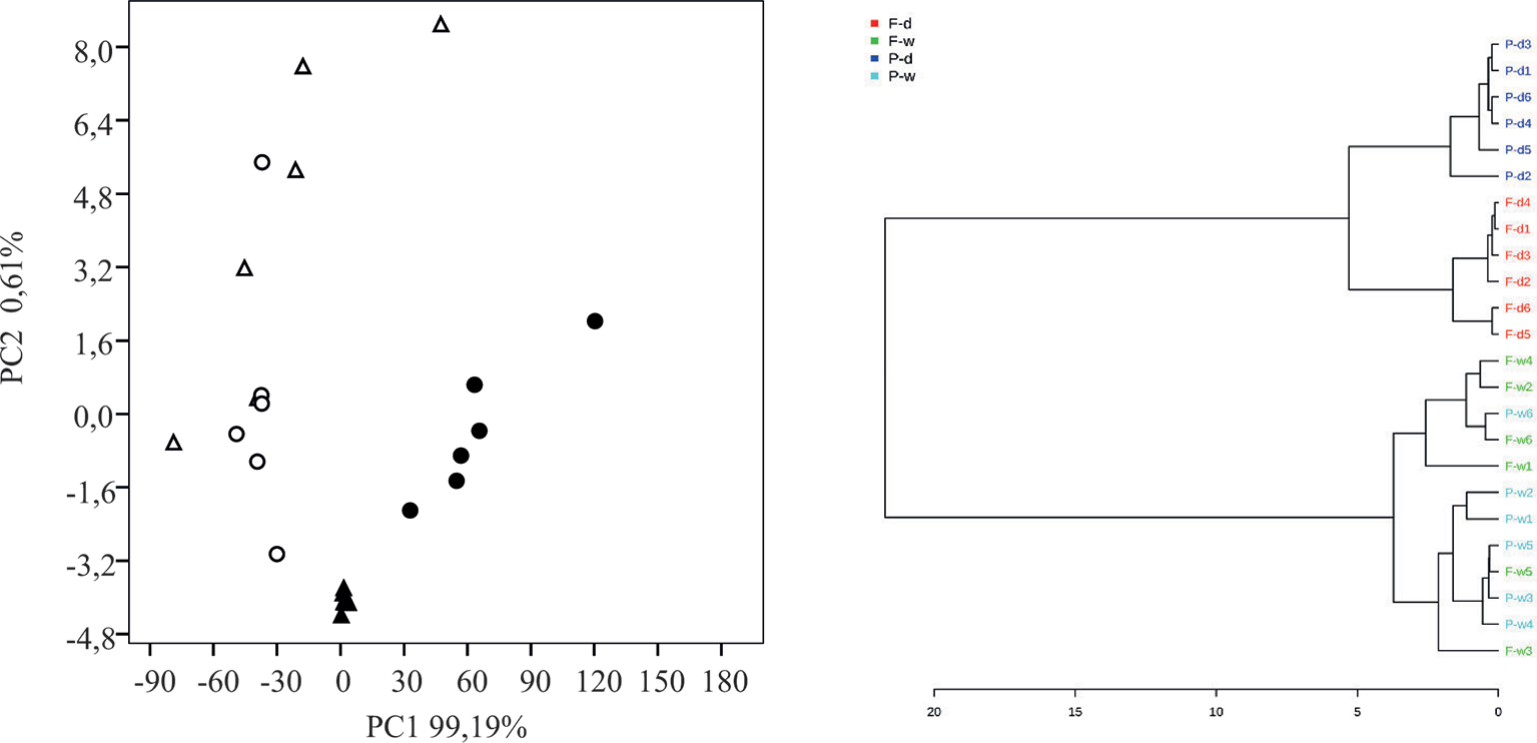
Multivariate analysis of morphological root traits from oat cultivar Patones and Flega droughted and well-watered plants. Principal component analysis (PCA) of nine root traits from seedlings growing in either pots or rhizotrons and adult plants from drought-susceptible Flega (triangles) and tolerant Patones (circles) well-watered (open symbols) and during water stress (solid symbols). PCA loadings related to this figure are available in **Supplementary Table 2**.

To compare further between seedlings and adult plants and to identify morphological root traits discriminating between susceptible and resistant cultivars, we performed PCA analysis with a total of nine root traits (total length, length of fine roots and surface area from seedlings growing in pots and rhizotrons and from adult plants growing in big containers) (**Figure 8**). The two first components accounted for more than 99% of the variance highlighting the reliability of the analysis. **Figure 8A** shows that well-watered oat plants clearly separated from those subjected to drought. Data showed no discrimination between the morphological root traits of well-watered plants indicating that differences between the two cultivars were induced by the water stress. Accordingly, drought-stressed plants separated in two groups. This separation between groups was confirmed by cluster analysis (**Figure 8B**). **Supplementary Table 2** shows that the main explanatory variables in PC1 were the length of adult plants (both total root length and length of fine roots) while the main explanatory variables in PC2 were the total root and fine root lengths from seedlings grown in pots. This suggest that the root morphological traits estimated from the pot-grown seedlings and adult plant experiments in container were equally informative and useful to discriminate between tolerant and susceptible genotypes.

## Discussion

Plant responses to water deficit may vary significantly according to stress duration and intensity, plant species, growth stage and method of application (Chaves et al., 2002; Jaleel et al., 2008). Consequently, many studies reported contradictory results regarding the effect of water deficit on plant growth and physiology. This occurs also frequently when the study only considers the drought responses of a single genotype, which prevents the differentiation between the mechanisms engaged early to cope with the stress, from those, engaged as ultimate survival mechanisms consequently to plant damage. For instance, proline has been proposed as a useful biomarker to identify drought tolerant plants since its accumulation is often induced by drought and it can contribute to stabilize membranes (Barnett and Naylor, 1966; Bates et al., 1973; Nayyar and Walia, 2003). However, in rice, sorghum and oat, accumulation of proline was shown to be a symptom of damage that correlated with the early decrease in cell membrane stability of susceptible genotypes, rather than an indication of tolerance (Ashraf and Foolad, 2007; Sánchez-Martín et al., 2015). These subtle differences cannot be inferred when only one genotype is assessed. Thus, reproducible and thoroughly documented experiments comparing tolerant and susceptible genotypes within a species are needed to robustly evaluate plant responses to various levels of water deficit (Aguirrezabal et al., 2006; Hummel et al., 2010; Sánchez-Martín et al., 2015).

To this aims, a series of experiments has been performed to characterize the root response to drought in two contrasting oat cultivars. These cultivars, were selected from an oat panel based on contrasting response to drought while exhibiting similar phenology and have been previously characterized at shoot level under controlled and field conditions (Sánchez-Martín et al., 2012; Sánchez-Martín et al., 2014; Sánchez-Martín et al., 2015; Rispail et al., 2018; Sánchez-Martín et al., 2018). As in previous work, here, no differences were detected between shoots of Flega and Patones grown under well-watered conditions. Under drought, however, Flega showed earlier and more severe aerial drought symptoms, in seedlings grown in pots and rhizotrons as well as in adult plants in container, suggesting that the water stress treatments were appropriate and induced similar responses. In addition, care was taken to monitor assiduously sRWC allowing direct comparison of cultivars under similar stress conditions.

Our results showed that, under drought, the tolerant cultivar Patones maintained higher root length, and root surface than the susceptible cultivar Flega in all experiments. This suggests that total root length and surface area are key morphological traits associated with drought tolerance, and higher productivity in adult plants, which is in agreement with previous reports (Comas et al., 2013). In addition, droughted Patones plants also showed higher branching rate and length of fine roots and lower average diameter and coarse to fine root ratio as compared with Flega in all experiments suggesting that tolerant plants responded to water deficit by stimulating fine root growth. Fine roots increase root-soil interaction and are the most active portion of the root system for water uptake. They also constitute the main component of total root length and surface area of herbaceous plants (Rewald et al., 2011; Comas et al., 2013) as confirmed here. Thus, increasing the length of fine roots has also been previously proposed as a key trait for water acquisition and productivity under drought (Wasson et al., 2012). For a given root biomass, smaller root diameters are related with longer roots and higher root surface, which increase not only the proportion of the root interacting with the soil but also the proportion of the root able to be colonized by mycorrhizal fungi. These fungi enhance uptake of immobile nutrients and improve leaf water and turgor potentials increasing the ability of the plant to cope with water stress conditions (Al-Karaki et al., 2004). In addition, higher number of fine roots increase root hydraulic conductivity by decreasing the apoplastic barrier of water entering the xylem (Eissenstat and Achor, 1999; Hernandez et al., 2010; Comas et al., 2012). On the other hand, increasing branching and hence the number of root tips, indicate continual root growth, which may be more important for the uptake of mobile resources than the total root length in itself, since the most active zones of water uptake are the young root tips (Haussling et al., 1988; Robinson et al., 1991). Altogether, the stimulation of root branching and fine roots development in Patones mediated its capacity to cope with the water stress conditions improving its drought tolerance as compared with Flega.

A prominent aspect of the study that deserves to be commented is the different growth rate observed between the experiments on seedling and adult plant roots under drought. We detected that the seedling root growth rate decreased under drought whereas it increased in adult plants as compared with control plants. In the literature diverse results can be found in this respect. Several studies reported a reduction of the root system under water deficit correlating with the stress severity in barley (Kage et al., 2004; Manivannan et al., 2007). By contrast, other studies on soybean and lupine reported a significant increase of root length during drought stress compared with well-watered plants (Hoogenboom et al., 1987; Rodrigues et al., 1995). Interestingly, Hoogenboom et al. (1987) reported that the drought-driven root growth stimulation was dependent on the plant growth stage at the time of stress imposition. Accordingly, drought stimulated root growth when it takes place at the end of the vegetative period and initial reproductive stages but not at the initial vegetative stages (Hoogenboom et al., 1987). These observations are in agreements with our results and provide an explanation for the differences in the root growth rate detected under drought between oat seedling and adult plants. Interestingly, irrespective of this, the trend of the tolerant genotype Patones was similar in both, seedlings or adult plant experiments in the sense that the root growth ratio during drought conditions was significantly higher than that of the susceptible genotype Flega. However, it seems that the stage of the plant at which water stress is imposed is crucial in the final absolute development of the root system. As reported previously by Palta et al. (2011), vigorous root systems at adult stages, may be useful under water-limited conditions, in particular in environments where crops rely largely on seasonal rainfall, such as the Mediterranean-type environments where there is a gradual depletion of water in the soil similar to that imposed in the adult plant experiment.

As stated above the great challenge for current breeding for drought tolerance is the accurate and fast phenotyping of root traits (Kuijken et al., 2015). Assessment of adult plant root system is more accurate as it is more directly correlated with productivity traits than that of seedlings. However, it is highly time-consuming for the elapsed time required to reach maturity and for the effort required to clean and scan large root systems. In addition, when plants are sown in the field it is difficult to assure the extraction of the whole root system, and in particular, of the finest and more fragile roots, which have a main role in coping with drought (Rewald et al., 2011; Comas et al., 2013). According to our data, the cultivation of plants in large containers under climatic field conditions may be useful for high throughput phenotyping. This approach might contribute to decipher the response to water stress under semi-natural conditions although it cannot take into account the soil structure and competition that may also contribute to final plant performance and hence definitive validation of plant growing under complete field conditions are desired. The rhizotron experiments allowed the continuous monitoring of many architectural and morphological traits of the roots through non-destructive methods. In our study the data collected from the continuous in-vivo monitoring of rhizotron experiments were strongly and significantly correlated with those obtained destructively at the end of these experiments and with those collected from pot experiments. Thus, in our hand, rhizotron experiments were highly informative and valuable confirming the usefulness of this approach to monitor root development in particular conditions such as in response to drought. Furthermore, for specific application of the stress, rhizotrons (where you can monitor root development non-invasively in different layers with different soil water content (see Nagel et al., 2015) may be the best choice. However, regarding the identification of tolerant and susceptible genotypes from PCA analysis, parameters recorded from rhizotron experiments had lower weight in the discrimination of drought tolerant and susceptible oat than those from seedlings grown in pots or from adult plants in container. Thus, for discriminating aspects, experiments carried out in seedlings grown in pots during gradual and slow water depletion combined with a strict monitoring of sRWC may be more convenient. In addition, despite the differences in absolute development observed between seedling and adult plant experiments, data from seedlings growing in pots were highly informative for discriminating between the tolerant and the susceptible genotypes, and, due to the rapidity and easy management of these experiments, these could be used as a first preliminary screening for high throughput root phenotyping. Since a correlation between all measured root traits was observed, any of them would be appropriate to identify drought tolerant oat genotypes.

Altogether, the different experiments carried out in the present work showed that the stimulation of fine root growth, are useful responses to cope with gradual soil water depletion in oat, both at seedling and adult plants. We also showed that although experiments in seedlings may not exactly mimic the response of adult plants they might be informative for discriminating between tolerant and susceptible plants and might facilitate phenotyping of large amount of samples, albeit it does not remove the need for validating these results under field conditions. In fact, we are now designing experiments to test the outcomes of our research in complete field conditions.

## Data Availability Statement

The datasets generated for this study are available on request to the corresponding author.

## Author Contributions

FC made most of the experimental work and data analysis. KN and CM supervised the rhizotron experiments and their results. NR and EP steered the research, designed experiments, and contributed to the interpretation of results and writing of the manuscript. All authors also contributed to critical reading and writing.

## Funding

This work was supported by the Spanish Ministry of Economy and Competitiveness [AGL2016-78965AGR], (AEI/FEDER, UE) and regional government through the AGR-253 group, the European Regional and Social Development Funds and the COST action FA1306. FC is holder of a FPI fellowship from the Spanish Ministry of Economy and Competitiveness.

## Conflict of Interest

The authors declare that the research was conducted in the absence of any commercial or financial relationships that could be construed as a potential conflict of interest.
