# Supplementary material for: Deciphering Root Architectural Traits Involved to Cope With Water Deficit in Oat
Source: Front Plant Sci. 2019 Nov 28;10:1558. doi: 10.3389/fpls.2019.01558 (PMC6892839; doi:10.3389/fpls.2019.01558)
Supplement: Supplementary file 1 [file DataSheet_1.pdf]

Frontiers in Plant Science

Deciphering root architectural components involved to cope with water deficit in oats

Francisco J Canales[1], Kerstin Nagel[2], Carmen Mülleri[2], Nicolas Rispail[1] and Elena Prats[1]

[1] *Institute for Sustainable Agriculture. Spanish Research Council (CSIC). Avda Menendez Pidal s/n. Cordoba 14080. Spain*
[2] *Institute of Bio- and Geosciences. IBG-2: Plant Sciences. Forschungszentrum Jülich GmbH. 52425Jülich. Germany.*

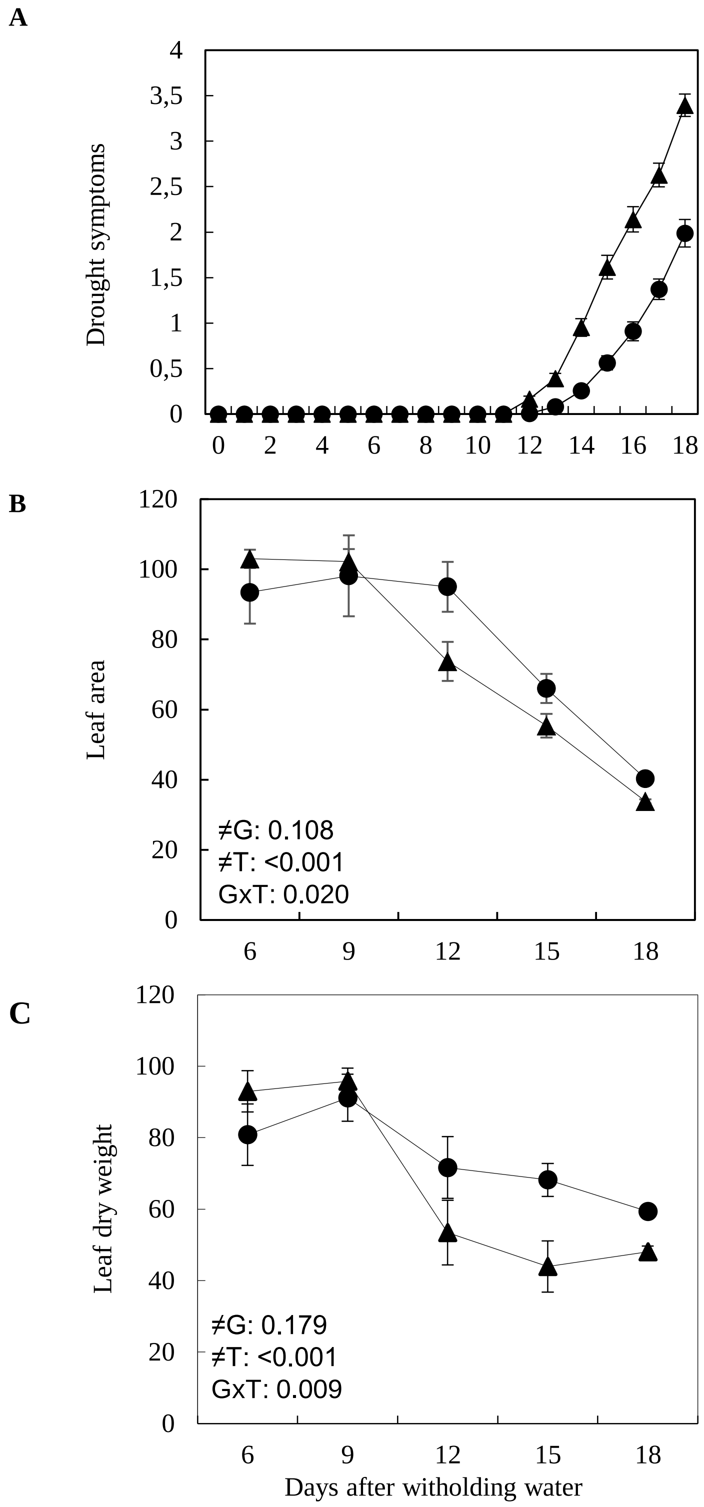

Supplemental Figure 1 Drought related traits assessed in shoots of seedlings growing in pots. A. Visible drought symptoms, B. leaf area and C. Total dry matter of Flega (triangles) and Patones (circles) droughted plants during a time course of water stress. Data are expressed as percentage respect to control plants growing in well-watered conditions and are mean of ten biological replicates + standard error

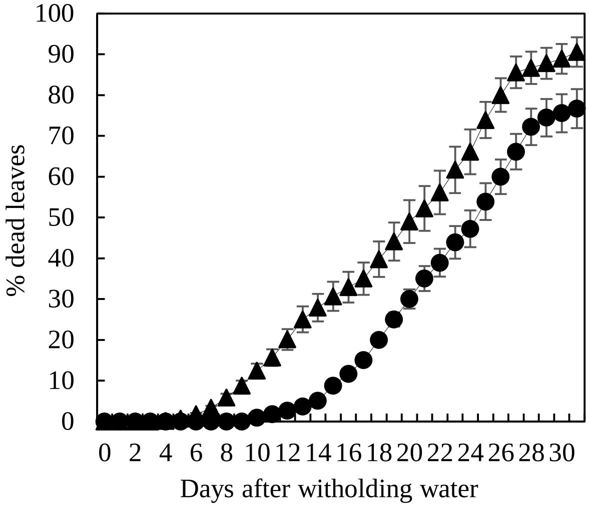

Supplemental Figure 2 Percentage of dead leaves of Flega and Patones adult plants growing under climatic field conditions during a drought time course. Data are expressed as percentage respect to control plants growing in well-watered conditions and are mean of eight biological replicates $\underline{+}$ standard error. ≠G, ≠T and GxT indicate statistical significance for the time course between genotypes (G), sampling times (T) and their interaction, respectively

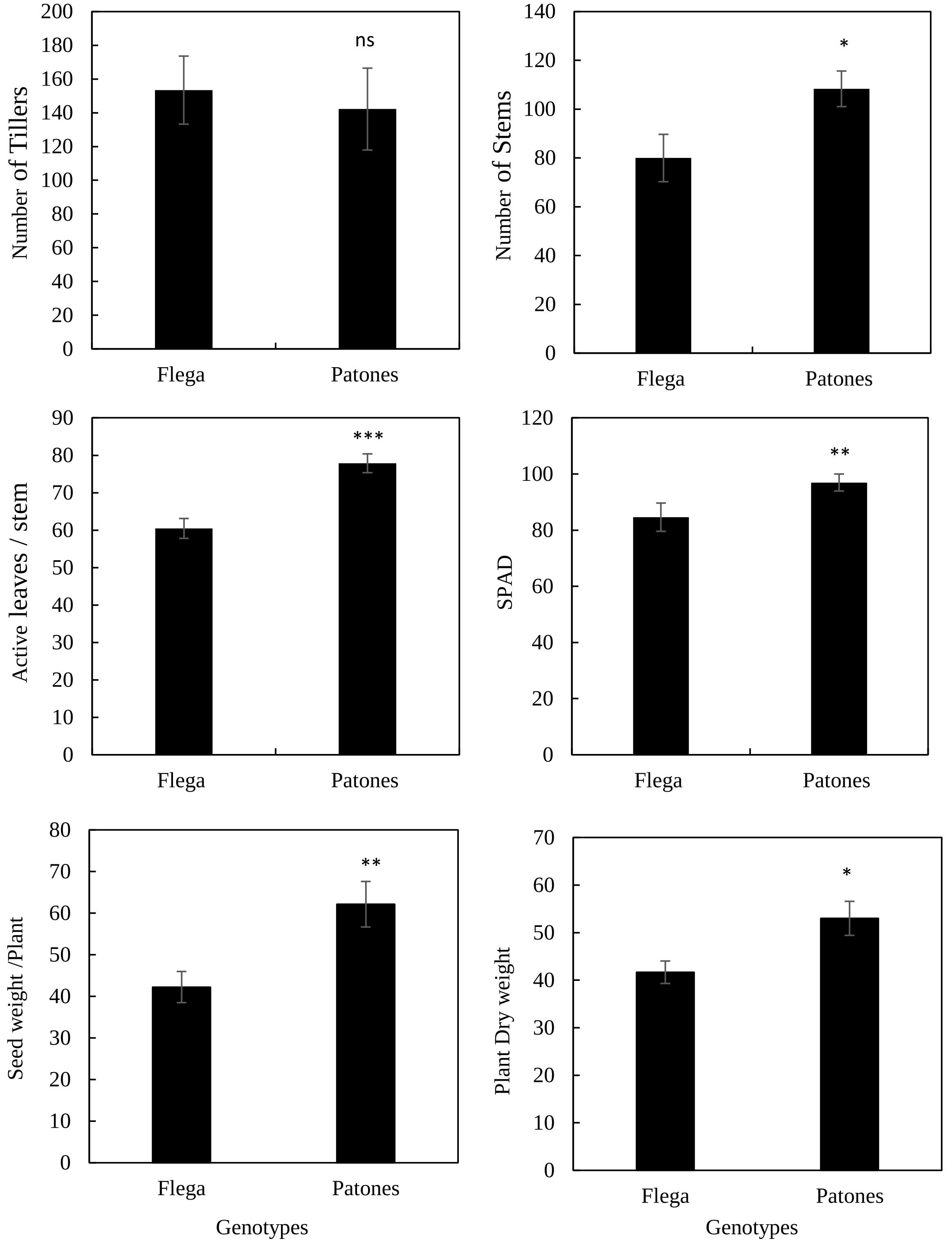

Supplemental Fig. 3 Effect of water deficit on growth, physiologycal and yield parameters of Flega and Patones adult plants under field conditions during a drought time course. Data are expressed as percentage respect to control plants growing in well-watered conditions and are mean of eight biological replicates ± standard error. *, **, *** indicate significant differences at $P<0.05$, 0.01 and 0.001, respectively.

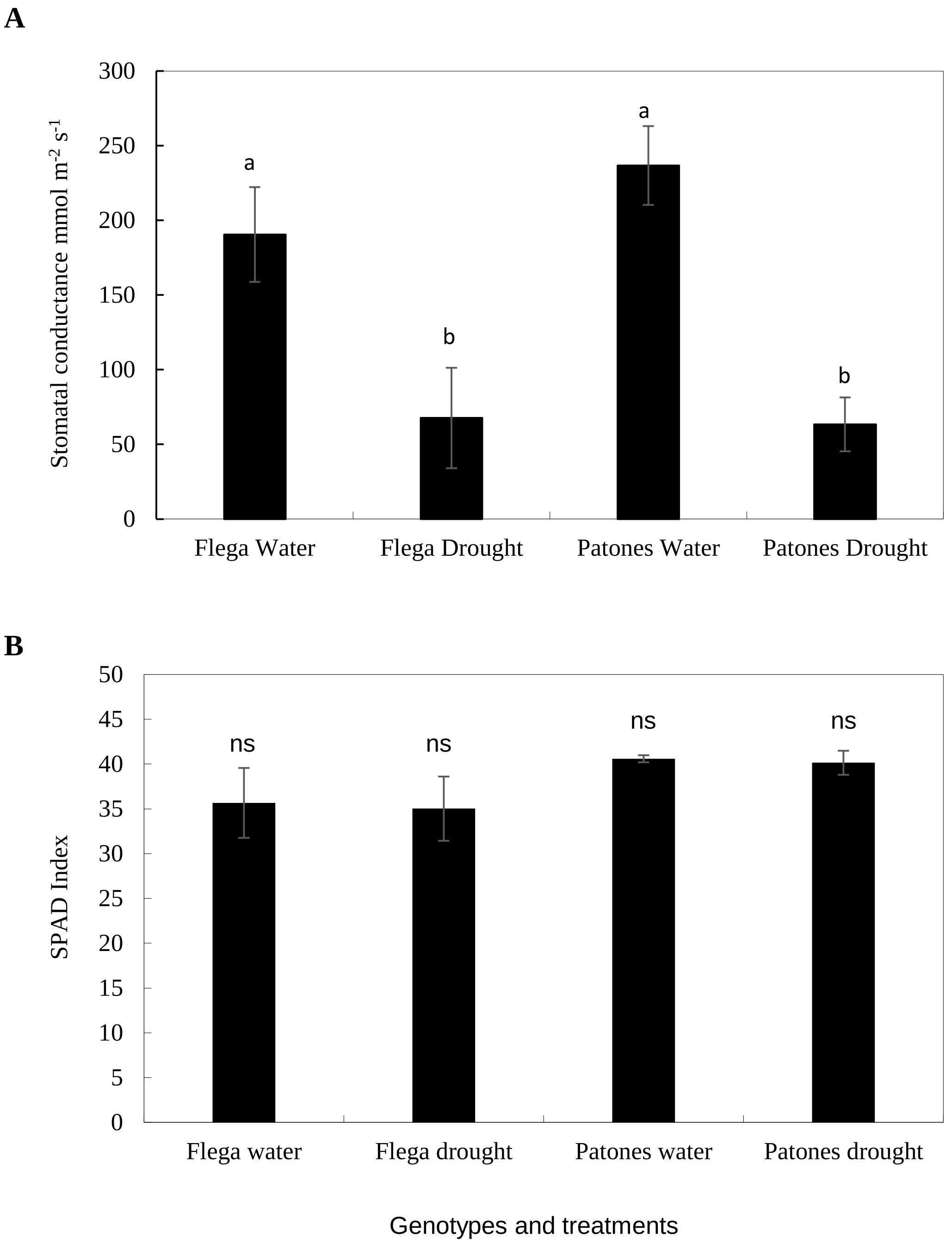

Supplemental Figure 4 A. Stomatal conductance and B. SPAD Index of flag leaves of Flega and Patones, well watered and exposed to water stress plants. For each plant the physiological parameters were measured in all stems and averaged. Data are mean of eight biological replicates $\underline{+}$ standard error. Different letters indicate significant differences at $P<0.001$

Supplemental Table 1. Shoot and root traits of well-watered Flega and Patones adult plants growth under climatic field conditions.

	**Flega water**	**Patones water**
	Shoot traits	
**Number of tillers**	4,75 ± 0,76	3,75 ± 0,22
**Number of stems**	15,50 ± 2,08	17,00 ± 2,56
**Active leaves / stem**	4,50 ± 0,30	4,61 ± 0,40
	Root traits	
**Total length (m)**	662,57 ± 203,69	726,40 ± 71,08
**Fine root length**	596,82 ± 206,08	680,38 ± 63,25
**Ratio coarse/fine**	0,08 ± 0,01	0,07 ± 0,00

Supplemental Table 2. PCA loadings of the variables on the first two axes. Numbers in bold indicate the highest coefficient for each axe. The root traits assessed are the total length, the root surface (Surf) and length of fine roots ranging between 0 and 0,5 mm (Fine), in seedlings growing either in pots (seed) or rhizoboxes (Rhi) or in adult plants growing under climatic field conditions (Adult)

	**Axis 1**	**Axis 2**
**Total root length (pot)**	-0,03025	**0,6873**
**Root Surface (pot)**	-0,002274	0,05298
**Fine roots (pot)**	-0,02904	**0,6555**
**Total root length (rhizotron)**	-0,00749	0,0935
**Root surface (rhizotron)**	-0,0007151	0,009676
**Fine roots (rhizotron)**	-0,005968	0,07298
**Total root length (adult)**	**0,7428**	-0,1016
**Root Surface (adult)**	0,06511	-0,1828
**Fine roots (adult)**	**0,665**	0,1931
